# A closer look at the role of apology in error disclosure: a simulation study

**DOI:** 10.3389/frhs.2025.1569550

**Published:** 2025-06-03

**Authors:** Sarah E. Shannon, Sherry Espin, Ben S. Dunlap, Lynne Robins, Peggy Soule Odegard, Carolyn Prouty, Sara Kim, Wendy Levinson, Cara Gray Helmer, Thomas H. Gallagher

**Affiliations:** ^1^ Mark and Robyn Jones College of Nursing, Montana State University, Bozeman, MT, United States; ^2^ Daphne Cockwell School of Nursing, Toronto Metropolitan University, Toronto, ON, Canada; ^3^ Department of Family Medicine, School of Medicine, University of Washington, Seattle, WA, United States; ^4^ Biomedical Informatics & Medical Education, School of Medicine, University of Washington, Seattle, WA, United States; ^5^ School of Pharmacy, University of Washington, Seattle, WA, United States; ^6^ Department of Public Health and Health Services, The Evergreen State College, Olympia, WA, United States; ^7^ Department of Surgery, School of Medicine, University of Washington, Seattle, WA, United States; ^8^ Department of Medicine, University of Toronto, Toronto, ON, Canada; ^9^ Washington State Hospital Association, Seattle, WA, United States; ^10^ Department of Medicine, Department of Bioethics & Humanities, School of Medicine, University of Washington, Seattle, WA, United States

**Keywords:** interprofessional, patient safety, disclosure, error, apology

## Abstract

**Background:**

The importance of open communication following harmful medical errors is widely accepted including the role of authentic apology. Yet, disclosure conversations remain difficult for clinicians and offering an authentic apology is challenging.

**Purpose:**

To better understand how clinicians can improve disclosures and apologies by using simulation to observe the approach clinicians use in the initial disclosure, where and when apologies occur within these conversations, what content apologies are linked with, who apologizes, and how apologies differ by their timing within the overall disclosure conversation.

**Methods:**

Forty-nine simulations of physician-nurse teams from the U.S. and Canada were videotaped planning and disclosing either a medical or surgical error to a patient-actress. Data from the disclosure portions were coded and analyzed using Atlas-Ti to describe the communication approach clinicians use when disclosing errors and the occurrence and timing of apologies within those disclosures.

**Results:**

Ninety-eight clinicians participated: 38 MD-RN teams from the U.S. and 11 from Canada. Of the 49 total simulated error disclosures, 30 involved medical teams disclosing an insulin overdose; 19 were surgical teams disclosing a lost specimen. The average length of the error disclosure conversations was 9.8 minutes (range = 6.1–14.2 min) and tended to follow a similar roadmap. On average, teams offered 2–3 apologies per disclosure (range = 0–9). These apologies occurred at all points during the disclosures and were offered by both physician and nurse participants.

**Discussion:**

Clinicians approached the initial disclosure conversations by addressing nine topics in a relatively consistent order. Apologies occurred throughout the disclosures. With opening comments, clinicians apologized to foreshadow bad news; with closing comments, they linked their remorse to broader professional and organizational goals around patient safety and transparency. Within the disclosure, clinicians sometimes linked the apology to their own emotional experience. More frequently, they linked apologies to the patient's emotional response, which may be more effective to ensure that patients hear that the clinicians' remorse is linked to patient suffering rather than clinician discomfort. To improve these difficult discussions, training materials and guidelines for communicating with patients after harm should reflect the complex role that apologies play.

## Introduction

At present, best practices in the United States and Canada for responding to harm events are encapsulated into what are known as Communication and Resolution Programs (1, 2). CRPs are comprehensive, systematic, and principled programs for preventing and responding to harm events in healthcare, and contain the following elements: early event identification; open and ongoing communication with patients and families about the harm event; event analysis and planning to prevent recurrences; support for all involved, and proactive financial and non-financial reconciliation (2). Harm communication conversations, sometimes referred to as “disclosures”, are complex verbal and non-verbal interpersonal interactions that include sharing information about what happened, supporting patient and family emotions, and discussing next steps.

At the center of these harm conversations with patients and their families is apology (3). In its simplest term, apology is understood as saying “I’m sorry.” The literature is clear that patients need and want to hear that health care providers are genuinely sorry for the harm event that occurred (4–9). For example, Mazor et al, surveyed 1,500 healthcare plan members in the U.S. about medical errors including their general attitudes around disclosure of errors (10). In this large study, nearly all participants endorsed having the error disclosed as soon as discovered (98.8%), learning what will be done to prevent similar errors (98.7%), receiving a full explanation (97.1%), having the disclosure be in-person (90.0%) and receiving an apology (87.6%).

Some scholars such as Lazare conceptualize an apology as including multiple elements in addition to an expression of regret, such as explaining how the problem occurred and committing to preventing recurrences (11). Other scholars such as Wailling situate a meaningful apology within a broad framework described as a restorative justice approach which combines relational and regulatory recommendations to guide organizations in supporting patients, families, clinicians and error investigators as they respond to harm in healthcare (12). Ramsey and colleagues discussed the concept of compounded harm which can occur for patients and their family members who have experienced a healthcare error when the disclosure and resolution process results in feelings of being powerless, inconsequential, manipulated, abandoned, de-humanized or disoriented (13). These findings underscore the importance of offering an authentic, meaningful apology while avoiding diminishing or discounting the patient and family experience.

While apologies have been shown to be associated with patient forgiveness following medical errors (7), there is a lack of evidence on the specific role that apologies play in the initial harm event conversations, thereby limiting the ability of recommendations and training programs to guide clinicians around how to approach these complex conversations and how to offer an apology most effectively. The purpose of this research was to use simulation to observe how initial disclosure conversations are structured by an interprofessional team of clinicians and where and when apologies occur within these conversations, including what content they are linked with, who provides the apology, and how apologies differ by their timing within the overall disclosure conversation. We used data from two linked research studies (one in the U.S. and one in Canada) involving physicians and nurses participating in a high-fidelity simulation of communicating about medical or surgical errors to patient actresses. The overall goal was to improve understanding of how clinicians structure a disclosure conversation and how they can effectively apologize following a harm event.

## Materials & methods

Data were analyzed from two, collaborative projects, one in the U.S. and one in Canada, designed to explore team-based error disclosure using simulation (14). Seven acute care organizations participated in the study (Table 1). In the U.S. Pacific Northwest, two organizations were large urban, university-affiliated, teaching hospitals. A third was a university-affiliated institution with a teaching mission, while the fourth was part of a health care cooperative. In Central Canada, all were teaching facilities; two were tertiary care settings and one was a community setting.

**Table 1:** Description of settings (*N* = 7), participants (*N* = 98), and simulated disclosures (*N* = 49).

SETTINGS	U.S.	Canada	Total
NUMBER	4	3	7
TYPE			
Tertiary care, teaching facility	2	2	4
Community, teaching facility	-	1	1
Federal, integrated care system	1	-	1
Cooperative care system	1	-	1
PARTICIPANTS			
NUMBER^a^	76	22	98
SPECIALTY^a^			
Medical clinicians	63% (48)	55% (12)	61% (60)
Surgical clinicians	37% (28)	45% (10)	39% (38)
TOTAL FEMALE % (*N*)^a^	63% (48)	59% (13)	62% (61)
FEMALE BY PROFESSION^a^			
Female MDs % (N)	37% (14)	18% (2)	33% (16)
Female RNs % (N)	89% (34)	100% (11)	92% (45)
YRS IN PRACTICE mean (s.d.)^b^	15.6 (11.8)		
YRS IN PRACTICE BY PROFESSION^b^			
MD mean yrs (s.d.)	12 (9.3)		
RN mean yrs (s.d.)	19 (12.8)		
SIMULATED ERROR DISCLOSURES			
NUMBER	38	11	49
TYPE OF ERROR DISCLOSED % (N)^a^			
Medical error	63% (24)	55% (6)	61% (30)
Surgical error	37% (14)	45% (5)	39% (19)
LENGTH OF SIMULATION			
Mean # of minutes (s.d.)^a^^,^^c^	9.7 (1.9)	10.1 (2.0)	9.8 (1.9)

^a^ Data from videotaped disclosures. N equals actual number of participants. Available for both U.S. and Canadian data.

^b^ Data from respondents who answered a separate web-based demographics survey. N does not equal number of participants in videotaped simulations due to non-responders. Web-based demographic survey not collected for Canadian simulation participants.

^c^ Length of disclosure measured from first “in-character” utterance to last utterance by either MD or RN participant.

The two research groups created vignettes describing errors common to medical or surgical care. Final vignettes were reviewed to insure generalizability and validity across the two geographic sites (Box 1). Recruitment methods included personal emails, referrals from organizational leaders, and the snowball approach.

BOX 1 **Description of Vignettes.**

**Medical Case: Insulin overdose**

Adult, female patient with diabetes admitted for exacerbation of chronic obstructive pulmonary disease (COPD). Physician handwrites “7U” rather than approved “7 units” on admission orders. Nurse mistook “7U” for “70”, checked with another nurse, and then gave 70 units of insulin. Nurse discovered patient unresponsive in room shortly thereafter. Patient successfully treated for hypoglycemia, spent the night in ICU, and is now fine. Patient is confused about why she became ill so quickly. Patient’s emotional response when error is revealed is distressed and sad.

**Surgical Case: Lost specimen**

Adult, female patient undergoes urgent surgery for vaginal bleeding. Suspicious cervical mass is removed, leading to significant perioperative bleeding. In the chaos, the mass is inadvertently discarded. Bleeding is controlled and the patient makes full surgical recovery. During the procedure, the circulating nurse was relieved by another nurse for a scheduled break. Following the procedure, the lab calls to report that the specimen container was received empty. An immediate search of the operating room confirms that the tissue specimen is irretrievably lost. The lack of biopsy will complicate diagnosis and decision making about treatment. Patient’s emotional response when error is revealed is distressed and sad.

For the error disclosure simulations, MD and RN participants were paired into medical or surgical teams, provided with the error vignette, and instructed to discuss the situation in preparation for speaking with a patient-actress. No other instructions were given to the MD-RN teams regarding how to approach the disclosure conversations. The MD-RN teams were joined by an actor scripted to be a standardized team member (STM). In the U.S. the STM was a hospital administrator; in Canada the STM was a nurse manager or pharmacist. The role of the STM was to ask questions during the team’s planning portion of the simulation to help the team verbally express their thinking (e.g., MD: “I’ll explain what happened.” STM: “What were you planning to say?”). During the disclosure portion, STMs were trained to allow the MD and RN participants to lead the disclosure, only contributing if prompted.

Following planning, the team disclosed the error to a patient-actress trained to respond in a standardized fashion. These simulated discussions represented the initial error disclosure conversation with a patient that occurs as part of the greater process of identifying, disclosing and resolving errors in healthcare. Actresses playing the patient had been coached to broach specific issues if the MD-RN teams did not voluntarily address these issues. Specifically, actresses were instructed to ask questions to clarify their understanding and to have an emotional reaction when it became clear that an error had occurred. If clinicians did not spontaneously offer reasons for how future errors would be prevented, patient-actresses were trained to ask about prevention of future, similar errors. Actresses were instructed to avoid making statements suggesting legal action (i.e., malpractice lawsuit) or other statements that might be construed as threatening. In the U.S., 5 different trained actresses portrayed patients. All were middle-to-older age women who had been trained to have a sad or scared emotional response upon learning of the error. In Canada, one actress portrayed both patient scenarios (medical and surgical). While the vignettes were identical to those used in the U.S., the Canadian group’s actress portrayed both an angry and sad emotional reaction. Teams were given the option whether to disclose the error as a team, dyad or by an individual member.

All simulations were videotaped. This analysis focused on the disclosure (not planning portion) of each team's simulation. Video data of each apology present in the U.S. and Canadian data were analyzed using complementary strategies. The analysis procedures were as follows. Analysis of the U.S. video data occurred first. The disclosure portion for each U.S. simulation was segmented into discrete conversational units by three experienced data coders who met regularly to achieve agreement on segmenting rules. Boundaries between conversational units were defined by changes in the overall topic of discussion as observed by the coders and based on examination of the pattern of conversations and what topics and themes were emerging throughout the simulation. Conversational units were coded using a detailed coding scheme developed by three U.S. coders (SS, BD, OD) including one experienced qualitative researcher (SS). Interrater reliability was verified at regular intervals following initial training. Explicit apologies and statements of clinician regret, remorse or sympathy were coded as an apology. After the initial, detailed coding was completed, the coding scheme was condensed into nine domain codes.

Data from the 11 Canadian simulations were analyzed using a method specific for the primary focus of this analysis. First, a Canadian researcher identified all instances of apology or statements of regret or remorse in the videos. Second, a U.S. researcher familiar with the prior segmenting procedures identified conversational units preceding and following each identified apology in the original video data. Third, these specific conversational units were coded using the final condensed domain coding structure (described above) by a Canadian researcher followed by verification by a U.S. researcher.

To be able to calculate the relative timing of apologies within the overall disclosure conversations, all simulations were normalized to 10 min in length (hereafter reported as “normalized minutes” or “n-min”). The start and end of each disclosure was defined as the first and last “in character” comments by the MD or RN. Fifty-two simulations were completed involving a physician, nurse, standardized team member, and patient-actress (U.S. = 40; Canada = 12); 49 were included in this analysis (U.S. = 38; Canada = 11). Two videos were excluded due to equipment problems and resulting data loss, and one due to incomplete recording.

Prior to data collection, the respective universities in the U.S. and Canada reviewed the research plans for protection of human subjects and approved all research procedures. In addition, the health care institutions where the research was conducted reviewed and approved all research procedures. All qualitative analyses were completed using ATLAS.ti qualitative analysis software (ATLAS.ti Scientific Software Development GmbH, ATLAS.ti Version 6.2. Berlin). All descriptive statistics were calculated using Stata 11 (StataCorp. 2009. *Stata Statistical Software: Release 11*. College Station, TX: StataCorp LP).

## Results

A total of 98 acute care physicians and nurses participated (Table 1). Almost two-thirds of the participants were from medical specialty areas vs. surgical. The majority of nurse participants were female (92%) and one third of the MDs were female (33%).

Of the 49 simulated error disclosures, 30 involved medical teams disclosing an insulin overdose while 19 were surgical teams disclosing a lost specimen. The length of the disclosure conversations averaged 9.8 min, ranged from 6.1–14.2 min and was similar between the two research groups with Canadian disclosures lasting slightly longer on average than U.S. disclosures (10.1 min vs. 9.7 min).

### How often do apologies occur during initial disclosure conversations and who offers an apology?

Teams generally offered at least one, and often more than one apology during the simulated error disclosures (Table 2). U.S. teams offered a mean of 3.5 apologies per disclosure while Canadian teams offered 1.8. In two disclosures (one U.S.; one Canadian), no apology or statement of regret or remorse was offered by any team member. In contrast, two U.S. teams offered nine apologies and one Canadian team offered four. Overall, there were 154 apologies within the 49 disclosures with MDs more likely to offer an apology (MD = 90; RN = 64). While female MDs represented only one third of physicians in this simulation study, they accounted for nearly half of the apologies offered by MDs with an apology rate of 2.6 per simulation while their male counterparts averaged 1.5. In this simulation study, medical teams averaged 3.6 apologies per disclosure while surgical teams averaged 2.4 apologies.

**Table 2:** Apologies in simulated disclosures: how often do they occur and who apologizes? (*N* = 154 apologies in 49 disclosures).

Disclosures	U.S.		Canada		Total	
Total simulated disclosures	38		11		49	
Total apologies	134		20		154	
Apologies per disclosure						
Mean	3.5		1.8		3.1	
Median	3		2		3	
Range	0–9		0–4		0–9	
Who provides apology?	MD	RN	MD	RN	MD	RN
Apologies by profession	2.0 (77)	1.5 (57)	1.1 (13)	0.6 (7)	1.8 (90)	1.3 (64)
Mean (N)						
Apologies by sex:						
Male	1.6 (38)	1.5 (6)	1.1 (10)	–	1.5 (48)	1.5 (6)
Female	2.8 (39)	1.5 (51)	1.5 (3)	0.6 (7)	2.6 (42)	1.3 (58)
Apologies by specialty:						
Medical team	2.2 (53)	1.9 (45)	1.3 (8)	0.5 (3)	2.0 (61)	1.6 (48)
Surgical team	1.7 (24)	0.9 (12)	1.0 (5)	0.8 (4)	1.5 (29)	0.8 (16)

### What is said in disclosures? at what point? by whom?

Based on detailed coding of the U.S. data, we found that teams approached initial disclosure conversations following a similar roadmap (Table 3). While specifics of the disclosure conversations varied, often in response to patients' specific questions, disclosure conversations generally covered a series of nine content domains in a relatively consistent order within the disclosure conversations. The content domains identified were (in order of appearance): openings, facts, responsibility, clinician emotion, patient emotion, policy, prevention, compensation, and closings.

**Table 3:** Content of simulated disclosures: What topics are included in disclosures, at what point, and by whom? (*N* = 38 simulations; 9 domain codes). U.S. data only.

Domain code	% Disclosures with domain code % MD vs RN speaker	Location of domain code within disclosure, either RN or MD speaking^a^
Openings:	100% disclosures (*N* = 38)	0.3 min (mean)
Introductions, inquiries about patient's welfare or recall of events, or a warning regarding content of the upcoming conversation	MD = 100% (38)	0.1 min (median)
	RN = 100% (38)	5.5 min (range)
Facts:	100% disclosures (*N* = 38)	2.6 min (mean)
Discussion of the events and/or facts surrounding the error; objective information	MD = 100% (38)	2.0 min (median)
	RN = 100% (38)	8.4 min (range)
Responsibility:	97% disclosures (*N* = 37)	3.5 min (mean)
Discussion of responsibility for the error including an individual, the team, and/or the system	MD = 95% (36)	2.9 min (median)
	RN = 82% (31)	8.9 min (range)
Clinician emotion:	68% disclosures (*N* = 26)	4.2 min (mean)
Discussion of MD's or RN's emotional reaction surrounding the error or disclosure of the error	MD = 53% (20)	4.0 min (median)
	RN = 37% (14)	9.1 min (range)
Patient emotion:	100% disclosures (*N* = 38)	4.8 min (mean)
Discussion of the patient's emotional reaction surrounding the error or disclosure of the error	MD = 100% (38)	4.9 min (median)
	RN = 84% (32)	9.7 min (range)
Policy:	37% disclosures (*N* = 14)	4.9 min (mean)
Discussion of patient safety policies or organizational mission related to patient safety	MD = 24% (9)	4.8 min (median)
	RN = 16% (6)	6.7 min (range)
Prevention:	100% disclosures (*N* = 38)	5.6 min (mean)
Discussion of prevention of future similar errors	MD = 95% (36)	5.6 min (median)
	RN = 63% (24)	7.4 min (range)
Compensation:	21% disclosures (*N* = 8)	7.2 min (mean)
Discussion of monetary compensation for harm from error	MD = 18% (7)	6.8 min (median)
	RN = 3% (1)	4.2 min (range)
Closings:	92% disclosures (*N* = 35)	7.4 min (mean)
Drawing conversation to a close, planning for follow-up, restating plan, saying goodbye	MD = 87% (33)	7.9 min (median)
	RN = 37% (14)	9.3 min (range)

^a^ All simulations were normalized to 10-minute duration to allow comparison.

Within these simulated disclosure conversations, MDs tended to start the disclosure conversations and to make more statements than nurses (MD = 66% of total codes vs. RN = 36% of total codes). In most cases, when a physician raised a topic, his or her nurse colleague would contribute verbally within that same content domain. Physicians were more likely than nurses, to discuss their emotions related to the error with just over half of MDs mentioning their emotional reaction (53%), while nurses commented on their own emotional experience in a little over a third of conversations (37%). Physicians were also more likely to discuss prevention of future errors, with 95% of MDs discussing the topic while 63% of nurses commented on error prevention. Similarly, in some conversations doctors raised the topic of compensation (18%), which was rarely discussed by nurses (3%).

### What disclosure content are apologies linked to and how do they differ by these linkages?

For both the U.S. and Canadian teams, apologies occurred at multiple points in the disclosure conversations but differed qualitatively depending on their timing within the overall conversation and the content the apology was linked with (Table 4).

**Table 4:** What content are apologies linked to in disclosures and how do apologies differ by these linkages? (*N* = 154 apologies in 49 disclosures).

Domain	% disclosures with apology in domain	Exemplar apologies associated with domain	Commentary
Openings: Introductions, inquires about patient's welfare or recall of events, or a warning regarding content of the upcoming conversation	10% (5) disclosures	Surgeon: “We think that's true [mistake was made] and we’re very sorry about it, because it presents a problem for what we’re gonna do about your care from this point on.”	Opening apology may alert patient to seriousness of the topic and suggests that the conversation that is about to occur will involve an apology.
		Physician: “I wanna explain to you, how this happened and I – we – want to offer our sincerest apologies as to what happened.”	
Facts: Discussion of the events and/or facts surrounding the error; objective information	47% (23) disclosures	Physician: “You ended up getting 70 units of NPH [insulin].” Patient: “That's what ha, happened to me?!” Physician: “That's what happened to you. I’m so sorry.”	Apologies linked only to facts; may sound brusque and uncaring for the patient.
		RN: “It [the specimen] was lost. There was a shift change. The nurses came in and we relieved each other, … we’re not sure exactly what happened to the specimen at this point. So, we actually don't have the specimen, and we’re sorry. …. We’re sorry about the fact that we don't have the actual specimen and we just wanted you to be aware.”	
Responsibility: Discussion of responsibility for the error including an individual, the team, and/or the system	76% (37) disclosures	Patient: “So, who's fault is this?” RN: “It's our fault, it's our fault, and we’re, we’re sorry.” Surgeon: “…And like Molly said, I’m very sorry that this has happened to you.”	Apologies linked to statements of responsibility may elicit reactions from the patient that the provider is primarily remorseful for their own experience.
		Patient: [To nurse] “I don't understand. You mistake “unit” for “zero”, how could you mistake “unit” for “zero"? Unit is a word.” Physician: “I think that, the order was not clearly written on my part, and, I think that, I think that was a mistake on my part and I apologize for that.”	
Clinician emotion: Discussion of MD's or RN's emotional reaction surrounding the error or disclosure of the error.	41% (20) disclosures	RN: “We found you unconscious almost immediately after you became unconscious, so we acted quickly. I just feel really sorry that it happened at all.”	Discussing the clinician's emotional reaction to the error may be appropriate in the disclosure, but an apology at this point links the regret to the clinician's experience rather than to the patient's.
		Surgeon: “I feel horrible. I’ve had this very sleepless night. I have never had something like this happen.”	
		Physician: “Both, I mean, all of us feel very, very sorry that this happened to you. It's not something that we set out to do. It's certainly not something that usually happens. It's something that we hope never happens, and for that, we apologize.”	
Patient Emotion: Discussion of the patient's emotional reaction surrounding the error or disclosure of the error.	94% (46) disclosures	Patient: “That's a pretty big mistake!” [hand to mouth in distress] RN: “You have every right to be upset, Susan. I just, I want you to know how sorry I am. I’m so glad you’re OK.”	Apologies linked to the patient's emotional reaction allow the patient to be the focus of the regret implied in an apology and are patient-centered.
		Patient: “I’m just,… this is really scary!” Surgeon: “It is. It's terribly scary.” RN: “Sure it is. (RN puts hand on patient's shoulder.) We’re so sorry that this has happened.”	
Policy: Discussion of patient safety policies or organizational mission related to patient safety.	24% (12) disclosures	Physician: “I'm so sorry for what happened.” RN: “I'm sorry too, but let me just tell you what we're doing so it won't happen again. Like the doctor said, they've implemented a hospital policy where you will not just use the letter “U” to represent units where it could be confused with a zero. It would always have to be spelled out. And, um, if I or any other nurses had any doubts we would immediately call the doctor to inquire whether it was “units” or “0s” so there wouldn't be any confusion…”	Apologies linked to statements about a policy may be unheard amongst details.
Prevention: Discussion of prevention of future similar errors	71% (35) disclosures	Surgeon: “Again, I just want to tell you, for all of us that we really did not mean for this to happen. I think we all feel very, very bad about it. We’re sorry that this happened to you. And we’ll do everything we can to make sure that, not only for you, that we can make this situation right, but also for other people in your situation, to come.”	Apologies linked to statements about preventing future errors may include statements about clinician remorse. Focusing on prevention is important but the apology may be unheard at this point due to the focus.
Compensation: iscussion of monetary compensation for harm from error	4% (2) disclosures	Physician: “We do want you to know that because the hospital system let you down, you’re certainly not going to pay for that mistake, so your extra days are not going to be…” Patient: [Interrupting] “I hadn't really thought about that yet.” Physician: “This is the least we can do. And… I’m sorry this happened.”	Apologies tied to discussions about compensation may not be heard by the patient as they focus on the details of what is being offered, or not offered.
Closing: Drawing conversation to a close, planning for follow-up, restating plan, saying goodbye	45% (22) disclosures	Surgeon: “So, you know, we wanna tell you how sorry and we wanna try to make it as right as we can for you.”	Apologies offered in closing are linked to statements about professional or organizational mission, values and ethics. These apologies may link the act of disclosure to these overarching professional principles or to core organizational values.
		Physician: “We’re so very sorry. Certainly we want you to trust the hospital again, and everyone here.”	
		RN: “Our deepest apologies for this error, and we just felt like the, the best thing to do was to tell you the, the whole story as best we could, and um, to tell you what your options are and to give you any more information that you need. So, I’m really sorry this happened.” Patient: “I’m really glad you did.” RN: “…We have a quality um, institution here and this unfortunately is, unfortunately is one of those human errors that rarely, rarely happen, but we just feel very sorry about it and wanted you to know exactly what happened.”	

Rarely, clinicians offered an apology in the opening portion of the disclosure. However, an apology at this point appeared to serve the purpose of alerting the patient to the type of conversation that was about to occur (i.e., one that would include an apology), to signal the tenor of the conversation (i.e., a serious conversation and one containing unpleasant information), or to express regret for the need for the disclosure conversation. In all of the simulations that included an apology in the opening portion, additional apologies were offered later in the conversation by the MD-RN team. An opening apology was generally brief and directed toward the upcoming conversation (“We want to offer our sincerest apologies as to what happened” or “I'm very sorry we're having to have this conversation”).

In nearly half of the disclosures (47%), clinicians included an apology during their factual explanation of the error. For example, in one disclosure of surgical error, the nurse explained how the specimen was lost and then apologized:

RN: “It [the specimen] was lost, there was a shift change. The nurses came in and relieved each other, there was a shift change and so we’re not sure exactly what happened to the specimen at this point. So, we actually don’t have the specimen, and we’re sorry.”

These apologies followed the basic formula of “X occurred and we’re sorry”, tended to be brief, direct, and almost brusque.

Most teams offered an apology when they discussed responsibility for the error (76% of all disclosures). These apologies often linked the clinician's remorse for the error to the circumstances surrounding the error, rather than to the harm experienced by the patient. For example, one physician discussed responsibility for the insulin error this way:

MD: “I think that when things like this happen we have a series of safety checks, and usually it’s a lot of things that fall apart before the error actually reaches a patient and causes harm. Definitely I have a role in it and I’m sorry for that.”

These apologies given in the context of conversations about responsibility could give the impression that the clinician was more focused on their own experience of having made an error rather than the patient's experience of being harmed.

Sometimes, clinicians offered apologies when they discussed their own emotional reactions to making an error or having the disclosure conversation. For example,

Physician: “[You are] doing well now and that makes me quite secure that you’re not going to have any long-lasting problems, or even hopefully short-term problems now from this. But it doesn’t take away how, how scared we are that this happened and how sorry that I am, and I know [RN] is, that this mistake was made.”

Apologies that were linked to the clinician's emotional experience rather than the patient's emotional experience, may suggest that the clinician's remorse was for his or her own emotional burden rather than for the harm or risk experienced by the patient.

In most of the disclosures, clinicians offered an apology or expression of remorse or regret when the patient's emotional reaction to the error was discussed (94%). Generally, clinicians simply named, acknowledged, affirmed, or supported the patient's emotional reaction (“It is. It is terribly scary” or “You have every right to be upset”). Often these apologies involved physical touch, such as taking the patient's hand or touching her shoulder. The exchange below by a medical team was initiated within the context of explaining what occurred and taking responsibility for the error, while linking the apology to the patient’s emotions:

PT: [reacting emotionally] “I'm sorry, this is just really scary, you know! I really try hard to take care of myself, and I have just this amount of control over this, and I come here, and you guys are professionals, and you're a doctor! Just because you wanted to write fast [you made an error]? I'm sorry, this is just scary for me.”

RN: “I understand.”

MD: “I understand.”

RN: “I understand, that it is scary to hear what happened and it must have been really scary to wake up in the ICU, and I'm so sorry for my part in it.”

The apologies that accompanied patient expressions of emotion tended to be unambiguously linked by the provider to the patient and her emotional experience rather than to the clinician's emotional reaction. These apologies were patient centered and allowed the patient to be the focus of the clinician's regret.

Apologies also occurred during the disclosure conversations when clinicians discussed policies, prevention of future errors, or financial compensation. These apologies tended to be buried within the detailed information that was specific to each of these topics and generally appeared almost as afterthoughts (e.g., “Make sure … we have a better safety check so that nothing like this happens again to you of anyone else again. And, I am deeply sorry” or “This [compensation] is the least we can do. And I’m sorry this happened”).

Finally, in nearly half of the disclosure conversations (45%), an apology was offered by either the MD or RN during the closing portion of the disclosure. Usually, an apology that was part of summarizing the disclosure conversation was the second or third apology offered in the overall disclosure conversation. In simulations that included an apology in the closing portion of the disclosure conversation, rather than apologizing specifically for the error, clinicians expressed regret for the patient's overall decreased quality of care or loss of trust, or affirmed their professional commitment to patient safety principles and transparency. (“And so again, I’m very sorry that this happened to you. … I hope that from this experience that we can learn how to make sure that this doesn’t happen again.”) Apologies included in the closing comments of the disclosure conversation tended to link the clinician's regret and remorse for the error to professional, organizational or ethical principles.

## Discussion

CRPs are increasingly recognized as the standard of care within the U.S. and Canada for responding when patients have been harmed by their healthcare (2). Healthcare organizations in the United States are now required by the Centers for Medicare and Medicaid Services to attest whether they have an evidence-based CRP and track its effectiveness using standard metrics. Yet the research done to date on patient experience with CRPs suggests considerable room for improvement exists in their implementation (15–17). Our study, which videotaped teams of healthcare workers communicating with standardized patients about errors, found that clinicians approached these difficult, high-stakes conversations using a similar roadmap. We also found that physicians and nurses offer apologies multiple times during intial error disclosure conversations, and that apologies at different points in the disclosure conversation serve different functions. The initial disclosure conversation is only the start of the CRP process which may involve multiple interactions with patients and families (12). An authentic apology at the beginning of the CRP process may set the tone for subsequent conversations. The path forward to improving the CRP process involves embracing the nuance and complexity of apologies within a common roadmap to guide clinicians in approaching initial disclosure discussions.

From childhood, most of us are taught that when something goes wrong you should be sure to say, “I’m sorry.” This research suggests a more complex reality. Clinicians often apologized multiple times throughout the course of error disclosure conversations, and those various apologies each served distinct communication purposes. Apologies occurring early in the disclosure conversation may be a warning, allowing patients and family members to emotionally prepare for bad news. Similarly, an early apology may alert the patient to anticipate an expression of humility, thereby heightening the patient's recall of an explicit apology that occurs later in the conversation. An apology given during the discussion or facts portion of the conversation, as occurred in almost half of error disclosures, may serve as part of an explanation for the error but may be unheard by the patient or family amidst the details being provided. In the majority of disclosures, apologies were provided during discussion of emotional reactions to the error. Most clinicians linked these apologies to the patient's emotional response rather than their own. Concluding apologies, on the other hand, particularly when they link the clinician's remorse to broader professional or organizational goals, may help to reestablish patient trust and affirm the shared values of honesty, patient safety and the patient's well-being. Developing best practices for communicating with patients and families after harm will be most effective if they can incorporate this more complex conversational reality.

The focus of harm conversations should rest squarely on understanding and addressing the needs and emotions of the affected patient and family (18, 19). However, just over 40% of clinicians in this study offered an apology focused on the clinician's own feelings and emotional reactions surrounding the error rather than the patient's. Clinicians often experience powerful emotions following medical errors including guilt over harming the patient, disappointment in their own performance, and anxiety about damage to their reputation (20). While it is natural as humans to seek forgiveness and focus on oneself, when clinician apologies center on the clinician's as opposed to the patient's experience, it may make the clinician's apology feel less authentic and sincere to the patient (5). Other studies on error disclosure have found a high importance on centering the patient's emotional experience in order to achieve an authentic apology (17). Clinicians should be trained how to focus the conversation on the patient's feelings and emotions, and away from their own emotions.

While much of the earlier literature in this field presented these discussions as involving an individual physician disclosing “their error” to a patient, more recent guidelines highlight how harm events generally result from the behavior of healthcare teams, and by extension frame harm event discussions as an interprofessional activity (21–23). However, our research highlighted considerable imbalance in how the nurses and physicians represented in this study participated in these discussions (24). In every conversational domain, physicians were more likely to speak and more likely to apologize than nurses. This dynamic is one that can lead to dysfunction in error disclosure if teams are not aware of it and do not plan around the different team roles in advance. By virtue of nurses' roles in the hospital, they are in the position to have regular daily interactions with patients giving them a unique perspective that physicians may not have. Given this role, nurses also may often be the first clinicians that patients and families come to with questions about errors. If nurses have been left out of error disclosure conversations and planning, they may be forced to answer evasively, leaving the patient and family feeling as if the hospital has something to hide. Teams involved in error disclosure should be aware of the various perspectives and benefits different clinicians bring to error disclosure conversations and should plan in advance to allow for this involvement (25). While the default on many teams is to allow physicians to lead the error disclosure, intentionally planning and shifting that dynamic may bring benefits for the team and the patients.

While CRP programs can use information from this study to inform new programs and practices around talking with patients and families about medical error, putting these techniques into practice also requires training. Anyone that engages in these error disclosure conversations should have training in order to learn the best practices that have been researched through studies such as this one and developed through CRP programs (26, 27). Training and practice should take place in a safe space, such as simulation, where mistakes can be made and corrected in order to get feedback for learning.

### Limitations

This analysis has several important limitations. First, standardized error vignettes were used in simulated situations rather than actual errors in real practice situations. This may differ from the way clinicians would present information in an actual case where more detailed factual and emotional information would be available to them. However, use of vignettes may control for variation in details and perceived severity of events and allow for replication of results. Second, the Canadian patient-actress presented a broader emotional range, specifically demonstrating anger, frustration and irritation, while the U.S. patient-actresses expressed sadness, fear and mild frustration. This difference may have affected clinicians' behaviors around apologies and the overall disclosure discussion. It is possible that the finding related to a slightly lower rate of apologies among Canadian teams is related to the actors' presentation of anger. Further research is needed to explore different patient emotional reactions. Third, the patient-actresses were trained to respond in a standardized way which likely impacted responses of the interprofessional team. Patient-actresses were coached to broach specific issues if the MD-RN teams did not voluntarily address these issues, specifically to ask questions to clarify their understanding, to have an emotional reaction upon learning an error had occurred, to inquire about prevention of future errors, and to avoid asking about legal recourse such as malpractice. In actual clinical practice, patients and families are likely to respond in variable ways not represented by the patient-actresses in this study. Future research is needed to explore variable responses by patients and families. Fourth, the U.S. and Canadian research groups used different procedures to process and initially analyze their respective data. To address this limitation, the U.S. team reviewed data coding of all Canadian data.

### Summary

Patients who have been harmed by their healthcare are in a uniquely vulnerable position. How healthcare professionals and organizations respond to these patients can either begin the process of rebuilding trust or serve to pour salt in the wound. The findings of this research offer a roadmap for approaching the initial disclosure conversation and highlight how an authentic and sincere apology goes far beyond merely uttering the words “I’m sorry.” Interprofessional teams preparing to discuss a harm event with patients should explicitly consider the multiple roles that apologies play in these interactions. They may wish to consider offering an apology in the opening portion of the conversation to prepare the patient for the type of conversation that is about to occur, followed by an apology when they discuss the impact of the error on the patient, and concluding with a statement that links their regret to a renewed commitment to patient safety and trust. Incorporating this more complex and nuanced understanding of the role of apology can help Communication and Resolution Programs better meet the needs of harmed patients and their families.

## Data Availability

The raw data supporting the conclusions of this article will be made available by the authors, without undue reservation.
